# Orthonasal bioactive volatiles and their effects on salivation: a pilot study

**DOI:** 10.1007/s00784-025-06689-4

**Published:** 2026-01-10

**Authors:** Wiktoria Potocka, Zainab Assy, Marja L. Laine, Floris J. Bikker

**Affiliations:** 1 Department of Oral Biochemistry, Academic Centre for Dentistry Amsterdam, University of Amsterdam and VU University Amsterdam, Gustav Mahlerlaan, 3004, 1081 LA, Amsterdam, the Netherlands; 2 Department of Periodontology, Academic Centre for Dentistry Amsterdam, University of Amsterdam and VU University Amsterdam, Gustav Mahlerlaan, 3004, 1081 LA, Amsterdam, the Netherlands

**Keywords:** Saliva, Dry mouth, Bioactive compound, Olfactory stimulus, Nasal inhaler

## Abstract

**Objectives:**

Previous findings showed that nasal exposure to mastic resin volatiles stimulated salivary flow both in healthy volunteers and dry-mouth patients. This prompted the search for new volatile sialagogic compounds as well as other, more standardized delivery methods. Therefore, this study aimed to assess the sialagogic effects of α-pinene, basil, eugenol, and guaiacol volatiles using nasal inhalers.

**Materials and methods:**

α-Pinene, basil, eugenol, and guaiacol volatiles were administered using nasal inhalers to act as an olfactory stimulus in healthy individuals (*n* = 12). Salivary flow, spinnbarkeit, and subjective mouthfeel were assessed before and after the use of the compounds. Furthermore, the possible effect of placebo nasal inhalers on salivary flow was also assessed.

**Results:**

Stimulation with basil and guaiacol applied with nasal inhalers resulted in an increase in salivary flow (*p* ≤ 0.01). Furthermore, basil improved the feeling of moistness in the oral cavity (*p* < 0.05). The use of nasal inhalers did not reveal any placebo effect in healthy individuals.

**Conclusions:**

These findings demonstrate that nasal inhalation of basil volatiles can effectively enhance salivary secretion and improve the sensation of oral moisture. This suggests basil in particular being a promising candidate for developing new topical treatment for dry mouth. Further studies with larger cohorts and clinical trials in dry-mouth patients are necessary to confirm and extend these results.

## Introduction

Chronic dry mouth commonly affects elderly and is more prevalent in women than men [1–3]. Dry mouth can be due to the subjective feeling in the oral cavity (xerostomia) or caused by an objective decrease in salivary flow known as hyposalivation [4]. Dry-mouth-related health problems lessen the quality of life (QOL) [5–8]. Although numerous dry-mouth relief products are available, many of them do not meet the patients’ needs [9–12], which results in non-compliance and further impairs the QOL.

Previously, it has been shown that many monoterpenes and monoterpene-rich compounds act as mild acetylcholinesterase (AChE) inhibitors in vivo, and by partly inhibiting the cholinergic receptor sites, they decrease acetylcholine (ACh) breakdown. This results in an increased and/or prolonged parasympathetic neurotransmission, and subsequently higher salivary flow. Further, it was found that ortho-nasal exposure to mastic resin volatiles caused sialagogic effects in healthy individuals and dry-mouth patients [13, 14]. Likewise, the scent of basil and guaiacol evoked comparable effects when delivered in form of nasal sprays [15]. This demonstrates that further research is required to develop novel treatments with the optimal standardised delivery methods.

Here, we aimed to assess the use of nasal inhalers containing either α-pinene, basil, eugenol or guaiacol in healthy individuals and focusing on the impact on the salivary flow, spinnbarkeit, perceived mouth feeling and scent pleasantness. Furthermore, the possible effect of placebo nasal inhalers on salivary flow was also assessed.

## Materials and methods

### Nasal inhalers

Nasal inhalers (YBMC, Raalte, the Netherlands) were used to deliver compounds. The inhalers were prepared by mixing either *Ocimum basilicum* (basil, CAS 8015–73–4, 84775–71–3), eugenol (CAS 97–53–0), guaiacol (CAS 90–05–1) or α-pinene (CAS 80–56–8) oils (Givaudan, Ashford, UK) and odourless Neutral Baby Oil (Unilever, London, United Kingdom) for a final concentration of 1% v/v. Basil oil consisted of: 51.18% linalool, 10.73% 1,8-cineole (eucalyptol), 7.15% eugenol, 3.29% cis-α-bergamotene, 3.29% β-elemene and other trace compounds (manufacturer provided data).

The cotton swabs of the inhalers were evenly soaked with a 200 µL of the 1% oil solution. Placebo nasal inhalers were prepared by soaking the cotton swab with a 200 µL of the Neutral Baby Oil.

### Participants

Twelve healthy volunteers took part in this study between June and July 2023 in Amsterdam, the Netherlands. The study was approved by the local Ethical Committee of the Academic Centre for Dentistry Amsterdam with protocol no: 2022–43674 and was carried out in compliance with relevant laws and institutional guidelines. The participants gave their written informed consent and were asked to answer a questionnaire. Healthy individuals ≥ 18 years were included. Exclusion criteria were: prescription medication (excluding contraception), systemic/autoimmune/chronic diseases, smokers, problems with olfactory perception, current flu, allergies to any of the compounds and/or products containing these compounds, current rhinitis and/or hay fever and pregnancy or lactating. On the day of the experiment, participants were asked not to wear any odorous products. For one hour before, participants were instructed to not eat or drink and not to brush their teeth or use oral care products. During the saliva collection, participants were asked to sit straight in a resting state, not to swallow, and not to move or talk.

### Placebo effect assessment

Six healthy volunteers participated to assess the possible effect of placebo nasal inhalers on salivary flow, and if the flow came back to the baseline levels after 10 min. Both unstimulated whole saliva (UWS) and stimulated whole saliva (SWS) were obtained by pooling the saliva on the bottom of mouth during the collection, and then spitting into the tube after the 5 min has passed. During the SWS collection, participants were asked to use the nasal inhalers by holding them underneath their nose (0.5–1 cm underneath the septum, at 60⁰ angle from the face) while inhaling normally, and alongside that they were asked to take one inhale actively into each nostril (deep breath) every 30 s (10 times total). The positioning of the nasal inhalers was demonstrated first by the researcher and eventually corrected when necessary.

Sampling was carried out in the morning (9:00–10:30 a.m.) due to variations in the circadian rhythm according to a previously described method [13–15].

1. UWS collection (5 min).
2. Break (1 min).
3. Placebo-SWS collection (5 min).
4. Break (10 min).
5. UWS-T10 min collection (5 min).

### Odour stimulation assessment

Saliva sampling was carried out in the morning as described above. Each compound was sampled on a different day (four days total). Participants received the compounds in random order, and were not informed beforehand what compounds they are receiving (single blinding). To ensure lack of expectation bias, the nasal inhalers were stored in closed tubes until the SWS collection.

Assessments took place in the following order:

1. “Before” questionnaire.
2. UWS collection (5 min).
3. Break (3 min).
4. Odour-SWS collection (5 min).
5. “After” questionnaire.

Spinnbarkeit measurements were carried out as described previously [15] using Neva meter (Ishikawa Iron Works Corporation, Tokyo, Japan [16]). Directly after the collection, saliva samples were homogenized by vortexing for 3 s. The Neva meter was set to “dry mode”, measure speed: 0.50 mm/s, dip depth: 0.95 mm, hold time up: 1 s, hold time down: 10 s and the detection method: optical. The measurements were carried out in quintuple using 80 µL of saliva. The average value (mm) was calculated by removing the highest and lowest readings and averaging the remaining three values.

Visual analogue scale (VAS) questions were set up on a 0–10 (cm) scale with responses recorded to one decimal point. Participants were asked to answer a question before and after the saliva collection: “How moist does your mouth feel at this moment?” – from “very dry” (0) to “very moist” (10). After the experiment, participants were asked to answer one more question: “How pleasant did you find the odour you just smelled?” – from “very unpleasant” (0) to “very pleasant” (10).

### Statistical analysis

GraphPad Prism (Dotmatics, Massachusetts, USA) was used to carry out statistical analysis. Shapiro-Wilk test (*p* < 0.05) was used to determine the normality of data. Change in salivary flow was calculated by: SWS-UWS. Differences in subjective mouth feeling were assessed by a paired t-test. Differences in salivary flow and spinnbarkeit were assessed by a paired Wilcoxon test. Pleasantness of scents and the placebo effect differences were assessed using one-way ANOVA with Tukey’s multiple comparisons test. Correlation between change in salivary flow and pleasantness was assessed using Pearson correlation coefficient (two-tailed, CI 95%). Values are reported as mean ± SD (normally distributed data) unless specified. P-values < 0.05 were considered statistically significant.

The effect size of the stimulation of salivary flow with basil (UWS vs. SWS) was calculated using G*Power 3.1.9.7. (Heinrich-Heine-Universität Düsseldorf, Düsseldorf, Germany [17]), with a result of 0.69. This effect size can be considered as moderate, indicating a meaningful impact of this intervention [18].

## Results

The use of nasal inhalers did not reveal any placebo effect in the six healthy individuals (F:4 M:2; 38.33 ± 14.68 years, range 22–63 years). The UWS was 0.30 ± 0.09 mL/min, placebo-SWS 0.31 ± 0.11 mL/min, and UWS-T10 min 0.28 ± 0.10 mL/min.

**Fig. 1:**
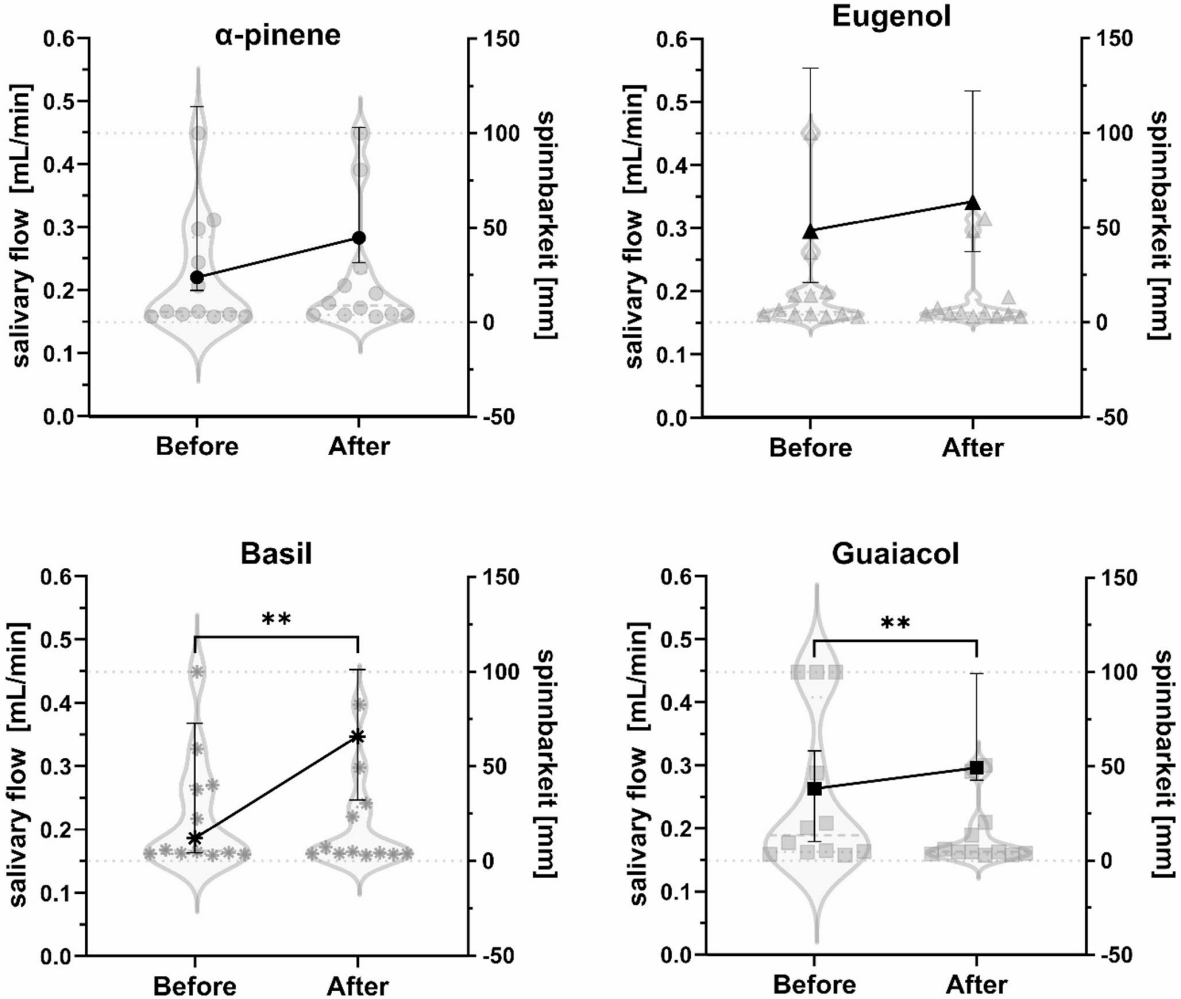
Salivary flow (mL/min) (left axis, line chart) and spinnbarkeit (mm) (right axis, violin chart) before (UWS) and after stimulation (SWS) with α-pinene, basil, eugenol and guaiacol by nasal inhalers in healthy participants (*n* = 12). Both values are expressed as median ± IQR. Paired Wilcoxon tests. **p ≤ 0.01 (salivary flow)

Stimulation with the volatile compounds using nasal inhalers was carried out in 12 healthy volunteers (F:7 M:5, 30.92 ± 6.19 years, range 23–43 years). There was a significant increase in salivary flow rates for both basil (median (IQR), UWS 0.19 (0.16–0.37) mL/min, SWS 0.35 (0.25–0.45) mL/min, *p* ≤ 0.01), and guaiacol (UWS 0.26 (0.18–0.32) mL/min, SWS 0.30 (0.28–0.45) mL/min, *p* ≤ 0.01) (Fig. 1). The salivary flow did not change after stimulation with α-pinene (UWS 0.22 (0.20–0.49) mL/min, SWS 0.28 (0.24–0.46) mL/min) and eugenol (UWS 0.29 (0.21–0.55) mL/min, SWS 0.34 (0.26–0.52) mL/min). There was no difference in spinnbarkeit after the application of any of the compounds: α-pinene (median (IQR), UWS 5.72 (3.73–44.97) mm, SWS 8.91 (3.86–26.42) mm), basil (UWS 5.42 (3.83–39.56) mm, SWS 4.64 (3.76–28.63) mm), eugenol (UWS 5.59 (4.15–15.70) mm, SWS 4.89 (3.44–12.01) mm) and guaiacol (UWS 13.52 (4.58–86.62) mm, SWS 4.71 (3.60-18.64) mm) (Fig. 1).

**Fig. 2:**
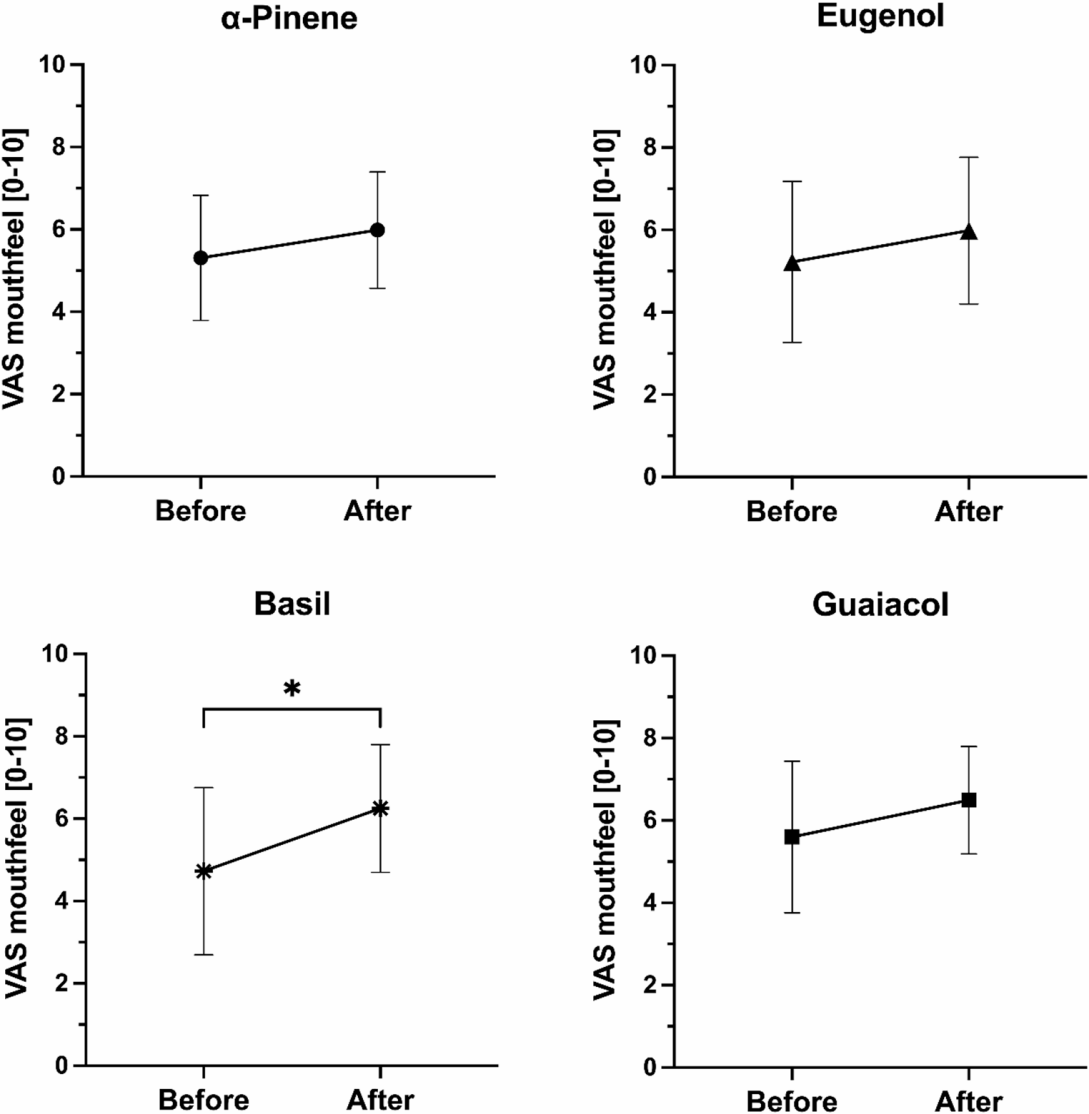
Subjective mouth feeling before and after stimulation with α-pinene, basil, eugenol and guaiacol by nasal inhalers in healthy participants (*n* = 12). The feeling was scored using visual analogue score (VAS) from 0 (“very dry”) to 10 (“very moist”) in response to a question “How moist does your mouth feel at this moment?”. Values are expressed as mean ± SD. Paired t-test. *p < 0.05

Along with increasing salivary flow, basil applied using inhalers also improved mouth feeling (before 4.73 ± 2.03, after 6.25 ± 1.55, *p* < 0.05) (Fig. 2). There was no difference after the use of α-pinene (before 5.31 ± 1.52, after 5.98 ± 1.41), eugenol (before 5.22 ± 1.95, after 5.98 ± 1.78) and guaiacol (before 5.60 ± 1.84, after 6.49 ± 1.30). α-Pinene was found to be the most pleasant (6.22 ± 1.54), which was followed by eugenol (6.07 ± 1.89), basil (5.51 ± 2.62) and guaiacol (3.93 ± 2.10). Guaiacol was perceived as significantly less pleasant than α-pinene (*p* < 0.05). There was no correlation between the change in salivary flow and pleasantness of any of the odours.

## Discussion

The goal of the study was to assess the salivary parameters after nasal stimulation with four volatile compounds using nasal inhalers in healthy individuals. The inhalers containing basil increased salivary flow and improved the perceived mouthfeel. Further, guaiacol also increased salivary flow, yet there was no change in the mouthfeel perception. Guaiacol was found the least, while α-pinene the most pleasant compound out of the four tested. These findings suggest that administering basil via nasal inhalers is the most suitable combination for further assessments aimed at developing a sialagogic therapy.

Previous results show that volatile compounds delivered ortho-nasally can increase salivary flow [13–15, 19–21]. The increment in salivation after the application of the inhalers is thought to be due to AChE inhibition at the muscarinic receptor site within acinar cells [22, 23], which amongst others (e.g. G-protein-coupled receptor (GPCR)-mediated intracellular calcium (Ca^2+^) signalling, transient receptor potential (TRP) channels), is one of the mechanisms regulating saliva secretion [24–26]. As reported previously [15], all the compounds applied here are mild AChE inhibitors in vitro. While basil was shown to be the weakest AChE inhibitor, the absolute change in salivary flow was the highest out of the four compounds tested [15]. We hypothesise that it might be due to basil oil being a mixed compound, and the other three being pure compounds. Basil oil consists primarily of linalool, 1,8-cineole, and eugenol. According to our previous findings [15] and literature [27, 28], 1,8-cineole and eugenol are moderate AChE inhibitors, yet linalool is not. However, it is worth noting that mixed compounds might contain molecules that bind other receptors playing a role in elevating salivary flow, such as TRP channels. Agonists of TRP melastatin 8 (TRPM8) and TRP vanilloid 1 (TRPV1) channels might indirectly increase salivary flow [19, 29, 30]. Two out of the main three basil constituents, 1,8-cineole and eugenol were identified as TRPM8 [31–33] and TRPV1 [34, 35] agonists. They might have a synergistic potential that is more suited for a sialagogue candidate than the pure compounds themselves.

On the other hand, guaiacol increased the salivary flow in participants, yet it was scored the least pleasant. At the same time, no correlation was found between the change in salivary flow and the perception of pleasantness, suggesting that trigeminal stimulation, rather than hedonic olfaction may be driving the effect [21]. In contrast, eugenol, known for its AChE inhibitory properties, did not evoke salivary response neither in this, nor the study using nasal sprays [15], which contradicts pharmacological expectations. The reason might be speculated to be inappropriate delivery vehicle or carrier solution chosen, or instability of the compound, and should be addressed in future studies.

Similarly to the outcomes of this study, mastic resin was previously reported to be a stronger sialagogue than pure α-pinene [14]. Yet, this study found that α-pinene, when delivered via nasal inhalers, did not significantly increase salivary flow or improve perceived mouthfeel. This contrasts with earlier studies where nasal inhalers containing α-pinene did show sialagogic effects [14]. The discrepancy is likely due to differences in concentration (1% in our study vs. 5% in previous studies), and thus suggests a dose-response relationship, which should be investigated in more detail. Moreover, further research is required to identify the mechanisms laying behind these physiological changes.

This study is a proof of concept – it draws a picture for a potential new therapy for dry mouth in the future, but it has to be noted that it is based on a small group of healthy volunteers. The next steps involve a larger volunteer group and later validation in patient cohorts, to accordingly assess the potency of basil oil as a sialagogue.

## Data Availability

The data that support the findings of this study are not openly available due to reasons of sensitivity and are available from the corresponding author upon reasonable request.
